# Raman-based sorting of microbial cells to link functions to their genes

**DOI:** 10.15698/mic2020.03.709

**Published:** 2020-02-10

**Authors:** Kang Soo Lee, Michael Wagner, Roman Stocker

**Affiliations:** 1 Institute for Environmental Engineering, Department of Civil, Environmental and Geomatic Engineering, ETH Zurich, Zurich, Switzerland.; 2 University of Vienna, Centre for Microbiology and Environmental Systems Science, Department of Microbiology and Ecosystem Science, Althanstrasse 14, 1090 Vienna, Austria.; 3 Center for Microbial Communities, Department of Chemistry and Bioscience, Aalborg University, 9220 Aalborg, Denmark.

**Keywords:** single cell Raman microspectroscopy, stable isotope probing, optical tweezers, microfluidic sorting, metagenomics, optofluidics

## Abstract

In our recent work, we developed an optofluidic platform that allows a direct link to be made between the phenotypes (functions) and the genotypes (genes) of microbial cells within natural communities. By combining stable isotope probing, optical tweezers, Raman microspectroscopy, and microfluidics, the platform performs automated Raman-based sorting of taxa from within a complex community in terms of their functional properties. In comparison with manual sorting approaches, our method provides high throughput (up to 500 cells per hour) and very high sorting accuracy (98.3 ± 1.7%), and significantly reduces the human labour required. The system provides an efficient manner to untangle the contributions of individual members within environmental and host-associated microbiomes. In this News and Thoughts, we provide an overview of our platform, describe potential applications, suggest ways in which the system could be improved, and discuss future directions in which Raman-based analysis of microbial populations might be developed.

There have been major recent technical advances toward understanding the composition of natural microbial communities and the contributions of individual organisms to their functioning. Stable isotope probing (SIP) provides a means to identify the organisms responsible for performing key functions within complex communities, and can provide information at the resolution of a single cell. Targeted sorting of cells from natural communities in terms of their functional properties and the use of the sorted cells for downstream DNA analysis or cultivation is becoming feasible, and makes it possible to establish a direct link between the phenotype (function) and the genotype (genome) of the members of a community. However, the approach is still in its infancy and major issues have precluded broader adoption, the most critical ones being the low throughput [1, 2] and the loss of cell viability induced during phenotypic identification [2, 3].

In our recent publication [4], we introduced a Raman-activated cell sorting (RACS) pipeline that enables microbial ecologists to link microbial metabolic phenotypes to their genotypes in a high-throughput and non-destructive manner (**Figure 1**). Within a complex natural microbial community, cells that are able to metabolize a particular compound can be labelled with deuterium (D) by incubating a community sample in a D_2_O-containing ‘minimal' (i.e., lacking nutrients) medium supplemented with the unlabelled compound of interest (e.g., glucose, mucin). After a short incubation, only active cells that were able to metabolize the compound of interest become D-labelled, resulting in the appearance of a signature peak in their Raman spectra (the carbon–deuterium ‘C–D' peak; the spectral region of 2,040–2,300 cm^-1^), which is easily detected using Raman microspectroscopy. By coupling SIP technology with an automated Raman-based sorting platform that uses optical tweezers and microfluidics to manipulate cells, we demonstrated that it is possible to achieve high-throughput sorting (200–500 cells/h; two orders of magnitude greater than by manual sorting) and the collection of D-labelled cells with high sorting accuracy (98.3 ± 1.7%). Our platform is suitable to sort individual cells for genomic analyses such as 16S rRNA gene sequencing and metagenomics, but also, since live cells are obtained, for cultivation and further ecological characterization or selection. We characterized and optimized the platform using laboratory strains of four model species (two intestinal, one soil and one marine – *Escherichia coli, Salmonella typhimurium, Bacillus subtilis*, and *Marinobacter adhaerens*). We then demonstrated the complete pipeline – from sample preparation to DNA analysis – to sort and identify mucin degraders within a complex community sample from the mouse colon. Our approach revealed that organisms belonging to the family Muribaculaceae, an abundant but under-characterized family present in the guts of homoeothermic animals, are key players in mucin degradation. Finally, we demonstrated the versatility of the system by sorting cells from a marine sample in terms of their cytochrome *c* signal. The few other cell sorting methods in the literature have applications limited to pure cultures (i.e., cells that can be cultured in the laboratory), large-sized cells (e.g., eukaryotes), and/or cells that contain a specific biochemical compound that generates a strong signal from ‘resonance' Raman scattering (e.g., carotenoids) [2, 5–9]. To the best of our knowledge, our platform is the first that works for diverse molecular signals in natural samples composed of microbial cells.

**Figure 1:**
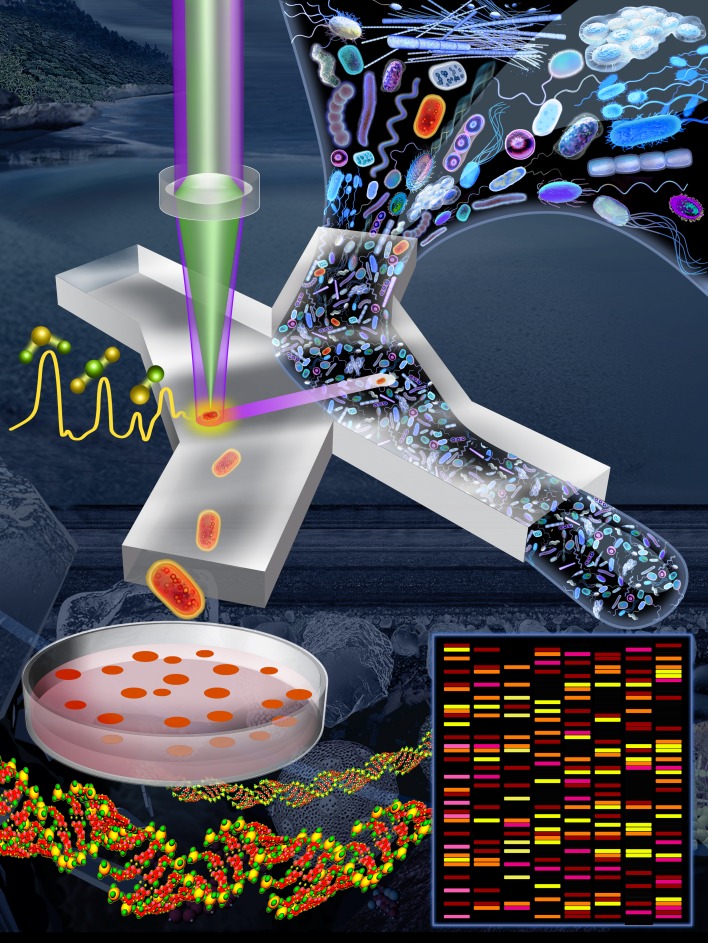
FIGURE 1: Pipeline to link functions of microbial cells to their genes. An environmental sample (e.g., marine, freshwater, soil, mammalian gut) is incubated for a short period in D_2_O-containing minimal medium (i.e., lacking nutrients) supplemented by a specific compound of interest (this initial step is not shown in this image). Cells able to metabolize that compound are active and so become labelled by deuterium (D), shown here in red. The sample is introduced into a microfluidic device in which single cells are captured in optical tweezers and analyzed using a Raman microspectroscope. The status of deuterium-labelled cells is apparent from a characteristic peak in their Raman spectrum. Deuterium-labelled cells (i.e., those with the metabolic property of interest) pass into a collection outlet and are available for subsequent genetic analysis (e.g., metagenomics, single-cell genomics) or cultivation for further phenotypic characterization. Image credit: Lee KS, Gorick G, and Stocker R.

A key parameter for successful cultivation of sorted cells is the laser power chosen for the Raman measurements and optical tweezers. However, recommendation of a universal laser power suitable for any application is difficult, because the magnitude of photophoretic damage depends both on the bacterial type [10], and on the wavelength used [11]. We used 532-nm (green) and 1,064-nm (infrared) lasers for Raman measurement and optical tweezing, respectively. For the Raman measurement, we employed a 15-mW laser (which yields 65.5 MW/m^2^ when using a 60× 1.2 NA objective; the photophoretic damage is dependent on the laser power density, not the power itself). This value was chosen by gradually changing the laser power to obtain the maximum power that did not cause lysis of an optically trapped cell over 10 s (sorting itself took a maximum of 7–8 s for each cell). Similarly, 400 mW was chosen for the 1,064-nm laser. Culture of sorted cells showed that they suffered no loss of viability. Empirical trials of this kind should be used to choose the power to employ for a given application.

The platform has many potential applications. For example, in host-associated microbiome research it provides a means to disentangle the symbiotic interaction between two partners (e.g., algae and associated bacteria). The host can be D-labelled by pre-culturing in D_2_O-containing medium, followed by a co-culture with the partner in non-D_2_O medium. Sorting the microbial partners in terms of D-labelling and subsequent DNA analysis can elucidate which taxa consume biomolecules from the host, and vice versa when the pre-culturing for D-labelling begins with the partner. In addition to classical deuterium probing, detection of those bacteria able to degrade particular compounds is possible by using labelled versions of those compounds: ^13^C-, ^15^N-, and D-labelled compounds can all be detected using Raman microspectroscopy. RACS thus provides a powerful means to determine the participants and the currency of symbiotic exchange. The same approach offers an efficient method to determine the bacteria performing important ecological or industrial functions, for example, using labelled dimethylsulfoniopropionate (DMSP) to detect DMSP-consuming bacteria within samples from the ocean, or labelled phosphate compounds such as Na_2_DPO_4_ to identify polyphosphate-accumulating organisms (PAO) in samples from wastewater treatment plants [12]. Furthermore, our sorting platform is extremely versatile: the spectral region in the sorting software can easily be tuned to use the presence of other functional molecules as the sorting criterion. Any molecule with a characteristic Raman spectrum is a potential target, for example, cytochrome *c* peaks (750; 1,127; 1,314; and 1,585 cm^-1^) as in our paper; calcium dipicolinate peaks (1,016; 1,400; 1,447; and 1,568 cm^-1^) to distinguish bacterial endospores from vegetative cells [13]; or the peak of surface-enhanced Raman scattering (SERS) nanotags used to label targeted pathogenic microbes [14].

Our platform can potentially be used to sort diverse microbes including algae (e.g., diatoms, cyanobacteria), fungi (e.g., yeast), and even mammalian cells (e.g., blood cells, tumor cells) that are larger than the bacterial cells used in our paper [4]. Raman microspectroscopy is based on a point measurement (i.e., the signal comes from the laser spot focused using an objective), and thus in general, the larger the cell (occupying more space in the measurement point), the greater the Raman signal intensity. The other crucial parameter is the optical tweezing efficiency (which determines the ability to hold the cell for the measurement period — key to reliable and reproducible Raman measurement). In general, the larger the cell, the greater the optical tweezing efficiency. Therefore, sorting of larger cells in our platform would be feasible. By the same considerations, there may be a lower limit on the applicable cell size when the optical tweezing force is not sufficient to capture the cell for the Raman measurement. We have successfully used the platform on cells down to a size of ~1 µ#x03BC;m, but the use of smaller microbes, such as viruses, may not be possible. Heterogeneity in cell morphology (e.g., spherical, rod-shaped, filamentous) is of lesser influence, because a cell of arbitrary shape is in any case trapped at the equilibrium position within the optical gradient force field [15, 16].

Improvements to the experimental setup could enhance its suitability to sort anaerobic samples, such as the mouse colon community in our work. For such samples, it is important that the platform maintains anaerobic conditions. However, in our experiment, even after being exposed to aerobic conditions in the gas-permeable polydimethylsiloxane (PDMS) microfluidic device and polyethylene (PE) tubing, the collected mouse colon taxa were still viable after sorting (unpublished data). This might be attributed to the brevity of the exposure (~1 h) to aerobic conditions, another advantage of rapid sorting. Nevertheless, the ability to maintain anaerobic conditions could be further improved by making the remaining components less gas permeable, such as by using polyetheretherketone (PEEK) or ethylene tetrafluoroethylene (ETFE) tubing, and the coating of the inner surface of the PDMS channels with gas-impermeable material (e.g., parylene C [17]) or by enclosing the whole device with acrylic, as in the anaerobic intestine-on-a-chip [18].

Future development of the platform could foster even wider applicability. A key feature in many applications is throughput, which is strongly related to the Raman signal sensitivity of the platform. For metagenomics, the collected cells after sorting are lysed and their DNA amplified by whole genome amplification (WGA). If the throughput were to be improved by 1–2 orders of magnitude (to a rate of a few tens of thousands of cells per hour), DNA amplification would no longer be necessary. Additional valuable information would thus be made available, such as the relative abundance of the taxa within a community; information that cannot be obtained after WGA. There is room for the necessary technical improvements. Methods employing ‘coherent' Raman scattering microspectroscopy, such as stimulated Raman spectroscopy (SRS), femtosecond stimulated Raman spectroscopy (FSRS), and coherent anti-Stokes Raman spectroscopy (CARS), provide powerful tools to increase the intrinsically weak spontaneous Raman scattering by more than thousand-fold. Fourier transform (FT)-CARS was recently used to detect astaxanthin productivity and photosynthetic dynamics of green algae in a microfluidic device that forms a single-file cell stream using a surface acoustic wave [19]. By avoiding the need to capture each cell for measurement, the device achieved a very high recording speed of cells as they passed in this flow (~2,000 events per second), and this technology would be applicable for Raman-activated sorting if two technical challenges were to be addressed. First, only large algal cells (*Haematococcus lacustris* and *Euglena gracilis*) were measured. There are many three-dimensional focusing techniques in microfluidics (e.g., hydrodynamic, acoustic, electric, and geometry-induced); however, it is still not an easy matter to tightly focus smaller bacterial cells (~1 µ#x03BC;m) in a narrow region of the flow. As the Raman signal is strongly dependent upon the position of the cell as it passes through the laser detection spot, the platform must be engineered to make tight focusing possible. Second, the existing system is a cell counter, and further engineering would be necessary to enable the platform to sort cells after the CARS measurement without greatly sacrificing throughput. In addition to these developments in the hardware, the software could also be made more versatile in the choice of the sorting criteria, by implementing machine-learning algorithms. In some situations, such as for cytochrome *c*–containing cells in marine community samples, cells show an inconsistent background in their Raman spectra depending on conditions (e.g., oxidation state), and thus universal sorting criteria are difficult to define by the user. In this instance, the software platform could first measure the spectra of an initial “learning” population of cells. It could then determine the sorting criterion by categorizing those data into a few sub-populations (one of which is the target), and then perform the actual sorting based upon this initial classification.

We anticipate that integration of our platform with recent technological advances in spectral data processing (e.g., machine learning) and electronics and photonics (e.g., coherent Raman system) will further improve its accuracy and throughput, opening new avenues for research in ecological, medical, and industrial microbiology. Already, armed with our RACS platform, researchers have a new efficient tool to understand the functioning of microbial communities.

## Abbreviations:

CARS: – coherent anti-Stokes Raman spectroscopy;
D: – deuterium;
DMSP: – dimethylsulfoniopropionate;
PDMS: – polydimethylsiloxane;
RACS: – Raman-activated cell sorting;
SIP: – stable isotope probing;
WGA: – whole genome amplification.
